# Supplementary material for: Immune-Cell-Derived Exosomes as a Potential Novel Tool to Investigate Immune Responsiveness in SCLC Patients: A Proof-of-Concept Study
Source: Cancers (Basel). 2024 Sep 14;16(18):3151. doi: 10.3390/cancers16183151 (PMC11430591; doi:10.3390/cancers16183151)

FIGURE 2C

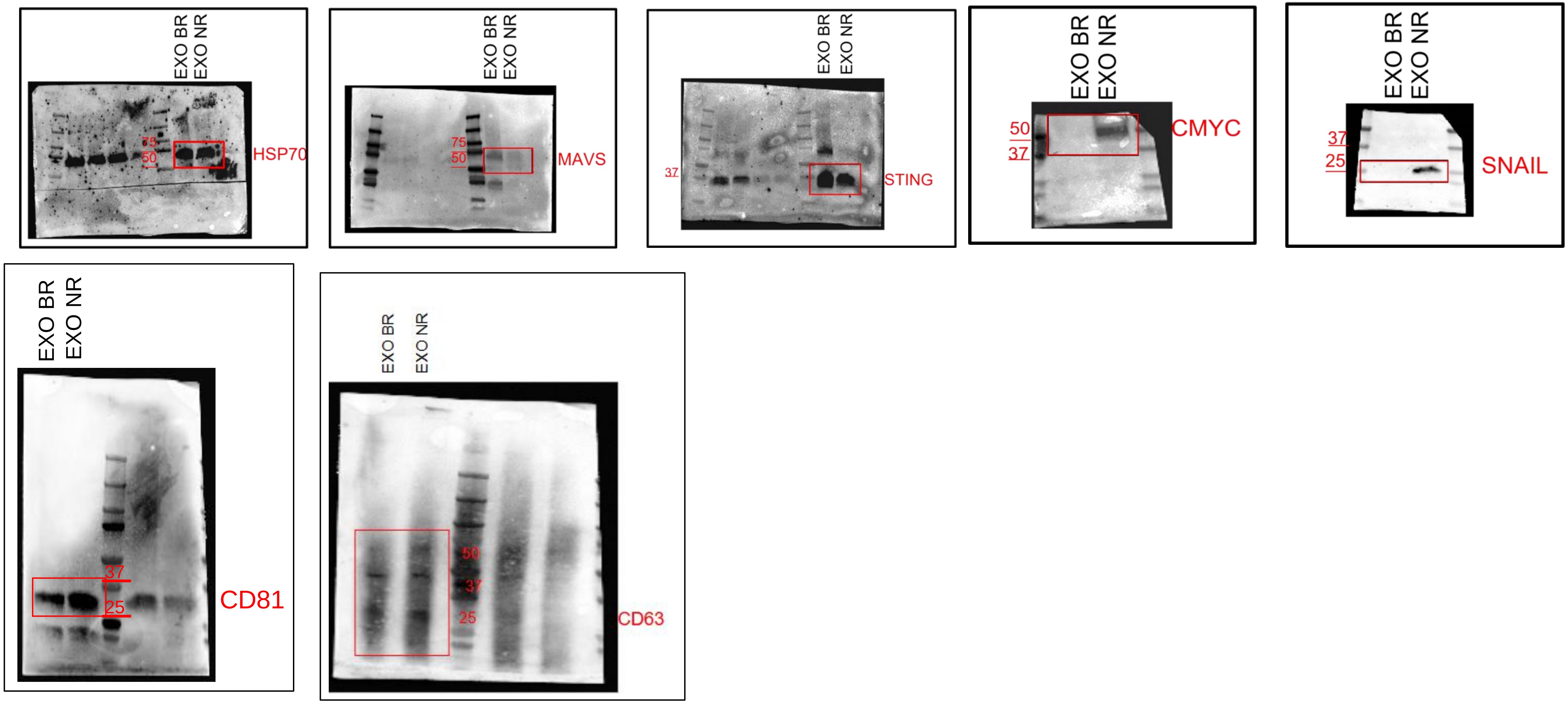
EXO BR
EXO NR
75
50
HSP70
EXO BR
EXO NR
75
50
MAVS
EXO BR
EXO NR
37
STING
EXO BR
EXO NR
50
37
CMYC
EXO BR
EXO NR
37
25
SNAIL
EXO BR
EXO NR
37
25
CD81
EXO BR
EXO NR
50
37
25
CD63

FIGURE 5A

left panel

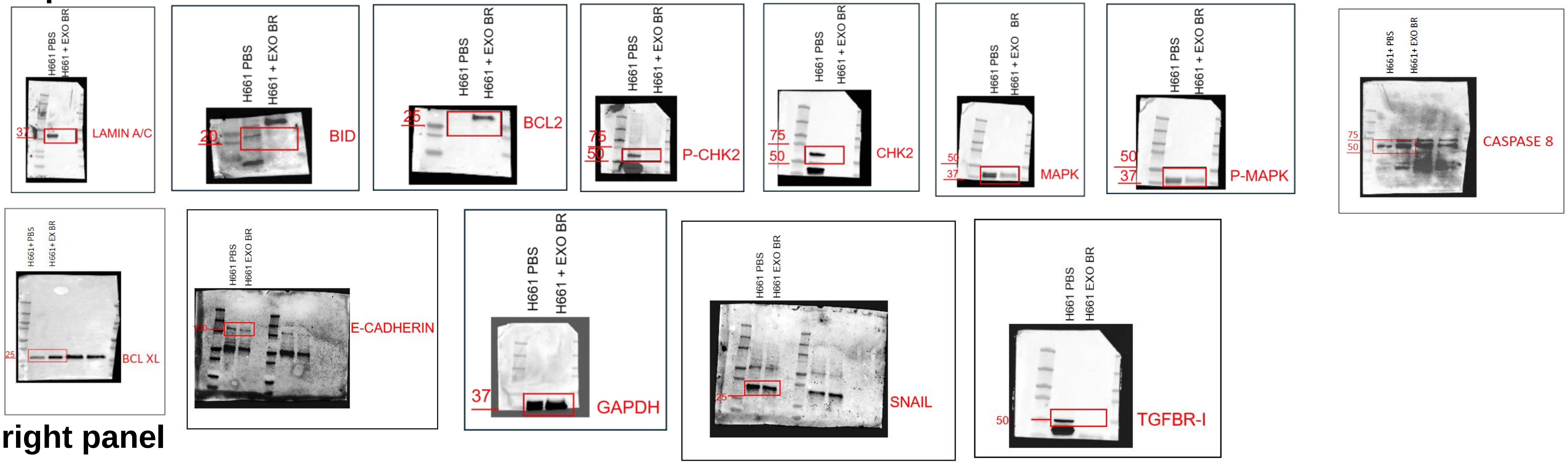

right panel

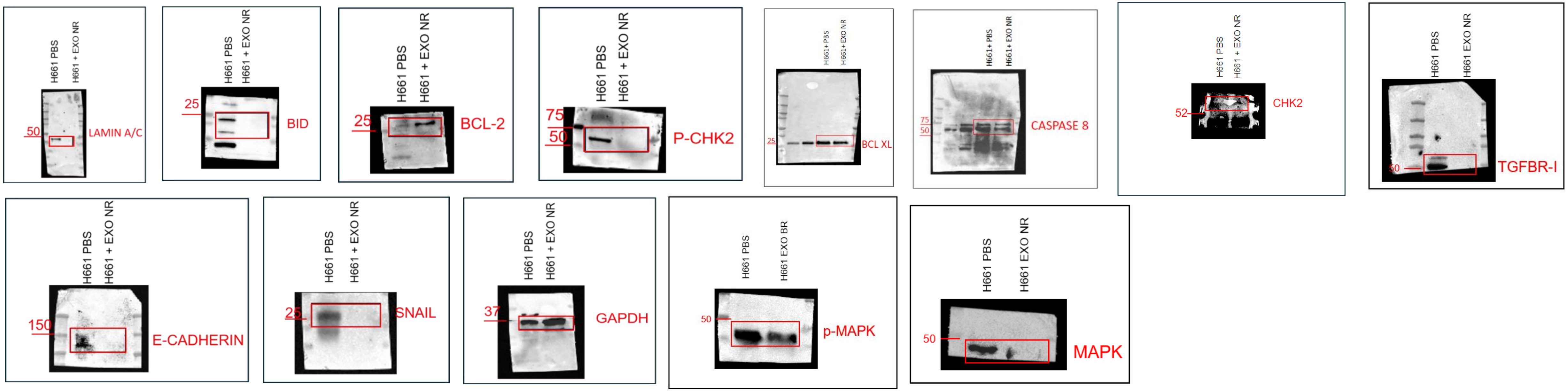

FIGURA 5B

left panel

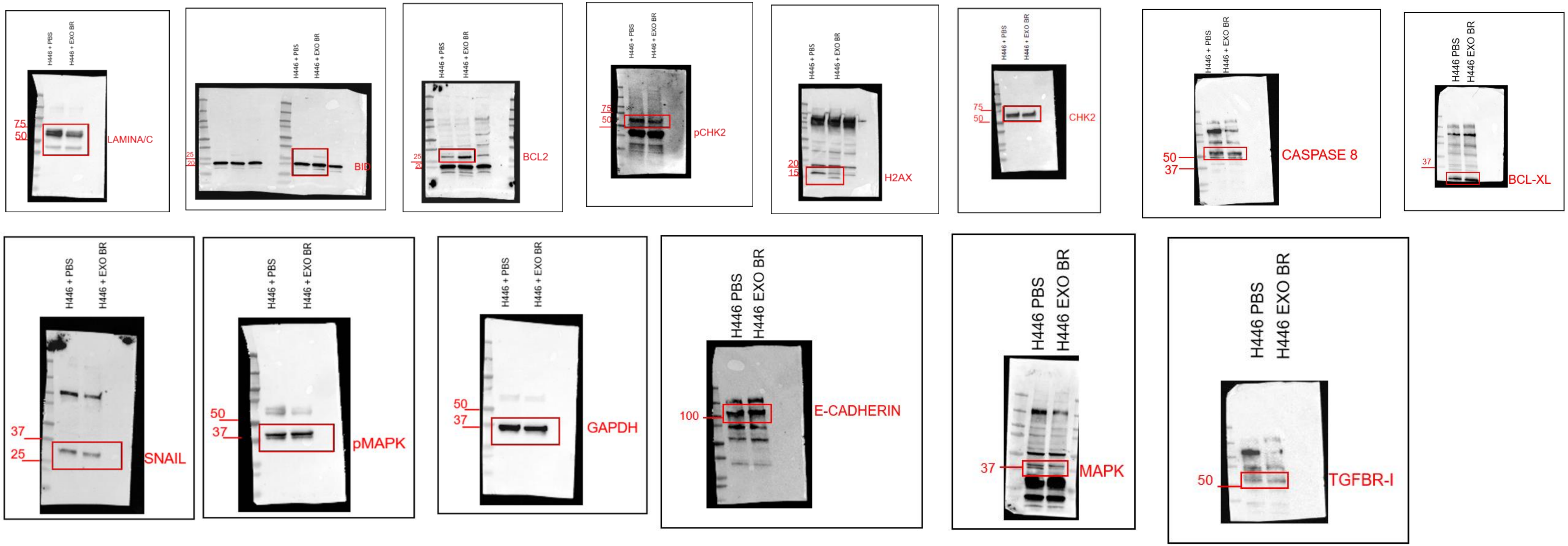

FIGURA 5B

right panel

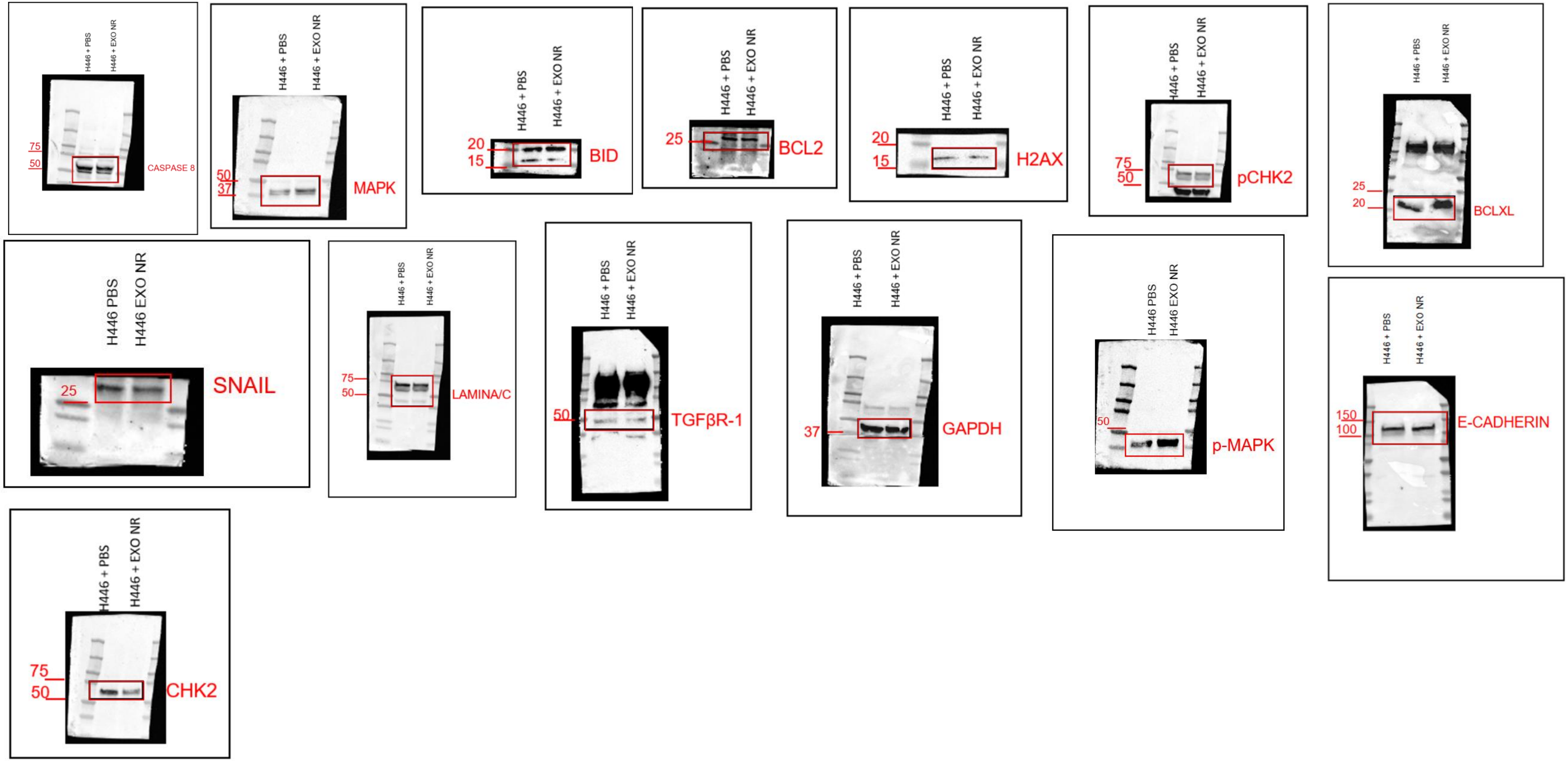

Supplementary S1

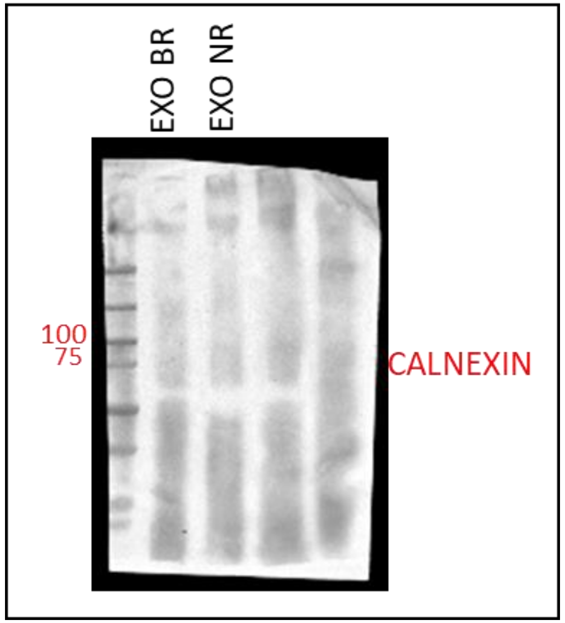

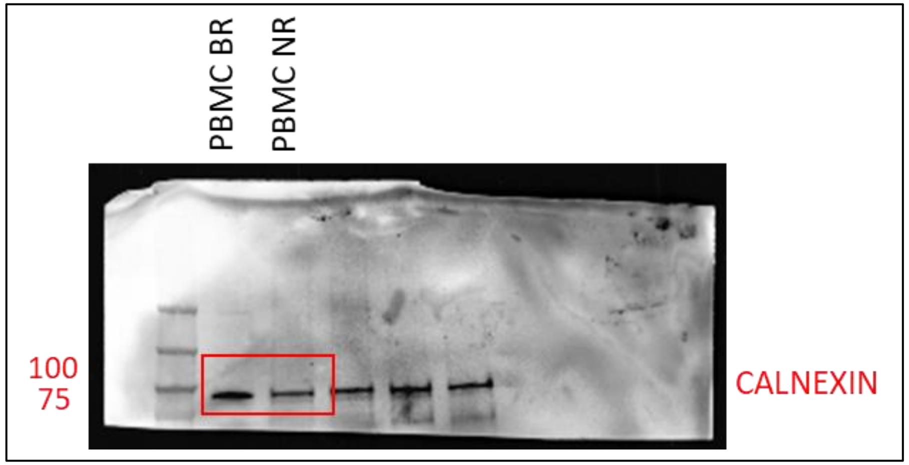

Supplement: Supplementary file 1 [file cancers-16-03151-s001.zip › cancers-3121510-File S1.pdf]
