# Supplementary material for: Immune-Cell-Derived Exosomes as a Potential Novel Tool to Investigate Immune Responsiveness in SCLC Patients: A Proof-of-Concept Study
Source: Cancers (Basel). 2024 Sep 14;16(18):3151. doi: 10.3390/cancers16183151 (PMC11430591; doi:10.3390/cancers16183151)
Supplement: Supplementary file 1 [file cancers-16-03151-s001.zip › cancers-3121510-supplementary.pdf]

Figure S1. Representative western blotting of whole cell lysates from (A) BR and NR PBMC from SCLC patients and (B) BR and NR PBMC-Exs showing expression of Calnexin (as negative exosomal marker). Ponceau S staining was used to ensure protein loading. At least three independent experiments were performed.

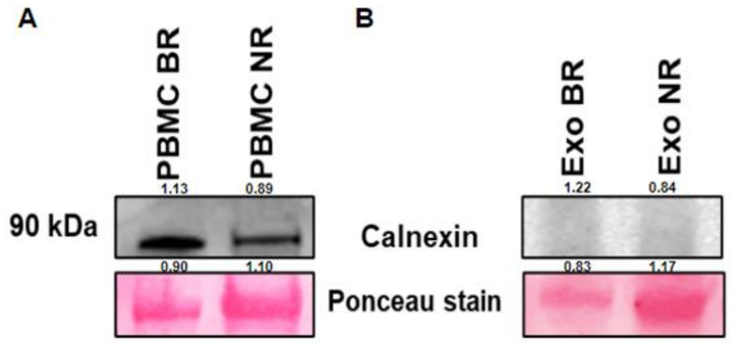

Figure S2. (A) Flow cytometry analysis of cell death by Annexin V/PI assay after co-culture for 24 h of PBMC-Ex from BR and NR donors. (B) Bar graph showing summary data of % Annexin V/PI positive cells; H661 (upper panel) and H446 (lower panel).

A

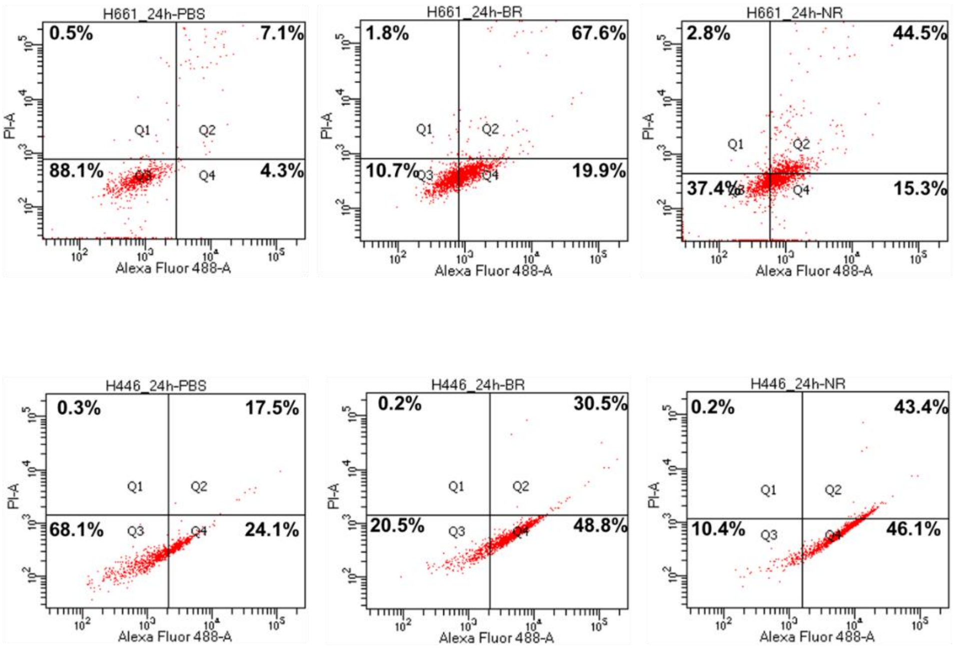

B

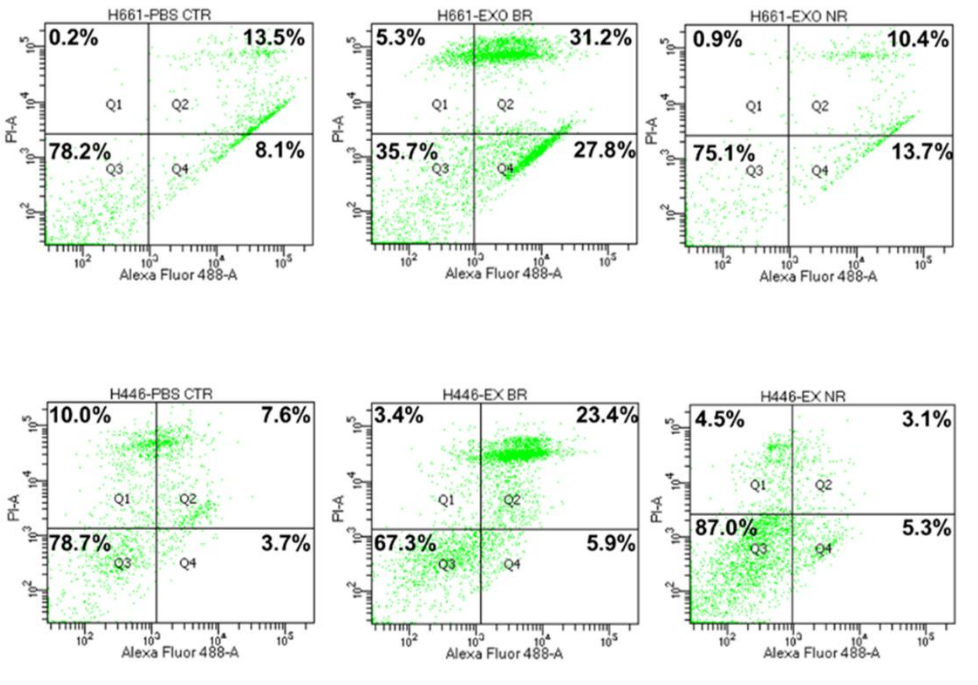

Figure S3. Semi-quantitative analysis of the results shown in Figure 5A and Figure 5B.

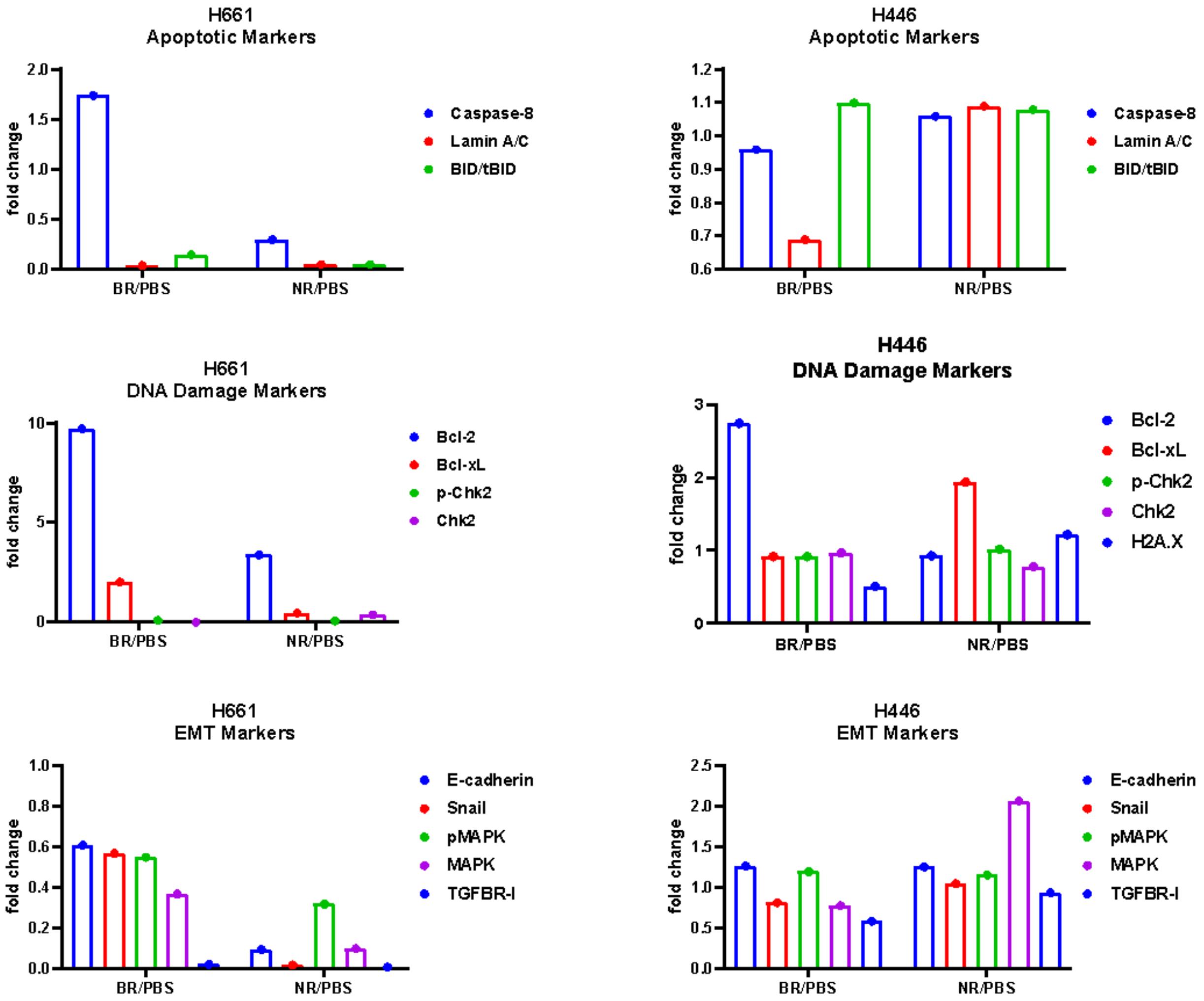

Table S1. Size distribution of the isolated PBMC-EXs.

	Concentration (particles/ml ± SD)	Mean (nm ± SD)
PBMC-EX BR	6.91E+11 ± 3.50E+11	135 ± 66
PBMC-EX NR	3.55E+11 ± 2.75E+10	220 ± 31
